# Initial nebulized budesonide versus a local off-label nebulized dexamethasone protocol in children with institution-defined grade III croup receiving epinephrine: a retrospective cohort study

**DOI:** 10.3389/fped.2026.1948559

**Published:** 2026-09-23

**Authors:** Yixian Yang, Lvping Xiong, Hong Liu

**Affiliations:** Department of Emergency Medicine, Jiangxi Provincial Children’s Hospital, Nanchang, Jiangxi, China

**Keywords:** budesonide, dexamethasone, epinephrine, inverse probability weighting, observational data, pediatric croup, propensity score

## Abstract

**Background:**

High-acuity croup requires prompt airway-directed treatment, but evidence comparing nebulized budesonide with a local institutional off-label nebulized-dexamethasone protocol is limited. This internationally unusual route is distinct from standard oral, intramuscular, or intravenous dexamethasone therapy.

**Methods:**

We conducted a single-center retrospective cohort study of children younger than 12 years hospitalized from 1 January 2014 through 31 December 2023. Pretreatment severity was fixed at the first institution-defined Grade III assessment. We compared initial nebulized budesonide with the local off-label nebulized-dexamethasone protocol among children receiving epinephrine within a prespecified ±15-minute treatment window; corticosteroid administration was time zero. The primary outcome was time to documented resolution of stridor at rest. Stabilized inverse probability of treatment weighting (IPTW) was winsorized at the 1st and 99th percentiles.

**Results:**

Of 12,480 all-grade croup presentation events, 452 charts underwent full review and 342 met institution-defined Grade III criteria. Three hundred children met all eligibility criteria (150 per strategy); 264 (88.0%) had institution-defined Grade III documented at the first assessment at our center, 36 (12.0%) progressed from Grade II after arrival, and 84 (28.0%) were interhospital transfers. The IPTW-adjusted difference in time to documented stridor resolution was −2.26 h (95% CI −2.63 to −1.89). Intensive care unit transfer occurred in 4/150 versus 6/150 children and intubation in 0/150 versus 1/150; these rare events do not establish equivalent safety.

**Conclusion:**

Initial nebulized budesonide was associated with faster documented recovery than the local off-label nebulized-dexamethasone protocol after adjustment for measured pretreatment variables. This comparison does not address standard oral, intramuscular, or intravenous dexamethasone therapy and should be interpreted as an adjusted observational association.

## 1 Introduction

Croup is a common acute upper-airway illness in children, characterized by barking cough, hoarseness, inspiratory stridor, and variable respiratory distress (1). Glucocorticoids improve symptoms and reduce healthcare revisits (2), while nebulized epinephrine provides rapid but transient relief in moderate-to-severe disease (1).

The 2023 Cochrane update included 45 randomized trials and 5,888 children. It confirmed the overall benefit of glucocorticoids but did not provide high-certainty contemporary evidence for the exact comparison evaluated here. Earlier randomized trials compared nebulized budesonide with oral or intramuscular dexamethasone, and a 2001 trial of mild croup found oral dexamethasone more effective than nebulized dexamethasone for subsequent care and symptom resolution (2–6). These routes and populations differ from the present local practice comparison.

More recent comparative evidence has addressed other corticosteroid questions: Parker and Cooper compared standard-dose dexamethasone, low-dose dexamethasone, and prednisolone in 2019; Jiang et al. compared dexamethasone with prednisone among hospitalized children in 2022; and D'Arienzo et al. examined one versus multiple dexamethasone doses in hospitalized croup in 2025 (7–9). Current guidance from the Canadian Paediatric Society and the Royal Children's Hospital Melbourne places oral dexamethasone within standard croup care, with parenteral corticosteroid used when clinically necessary, and nebulized epinephrine for moderate-to-severe disease; neither presents nebulized dexamethasone as a standard route (10, 11). No eligible contemporary study directly comparing nebulized budesonide with nebulized dexamethasone was identified in the targeted, non-systematic search described in Supplementary Methods S1. Thus, the exact comparison remains uncommon. Here, the comparator was a local institutional protocol using off-label nebulization of dexamethasone sodium phosphate injection; nebulization was not an approved route in the product labeling and must not be conflated with standard oral, intramuscular, or intravenous dexamethasone.^1^

Treatment allocation in routine care may reflect recorded severity, calendar time, referral status, drug availability, clinician preference, parental concern, bed availability, and undocumented clinical appearance. Propensity-score methods can balance measured pretreatment variables but cannot remove unmeasured confounding. In particular, if clinicians preferentially selected dexamethasone for children who appeared sicker in ways not captured in the record, the apparent budesonide advantage could be exaggerated; supply or protocol changes could bias the estimate in the opposite direction.

We therefore aimed to compare initial nebulized budesonide with the local nebulized-dexamethasone strategy among hospitalized children receiving epinephrine and meeting institution-defined Grade III criteria, while explicitly defining cohort entry, time zero, treatment timing, overlap, missingness, and the limits of causal interpretation.

## 2 Materials and methods

### 2.1 Study design, setting, and reporting

We conducted a single-center retrospective cohort study at Jiangxi Provincial Children's Hospital, Nanchang, China, from 1 January 2014 through 31 December 2023. The hospital is a provincial tertiary pediatric referral center. Reporting followed the STROBE and RECORD statements (12, 13).

### 2.2 Ethics

The Ethics Committee of Jiangxi Provincial Children’s Hospital approved the study (JXSETYY-YXKY-20250027) and waived individual informed consent because the study used anonymized routinely collected retrospective data.

### 2.3 Source population and cohort assembly

Croup presentation events were identified across emergency and inpatient records using a prespecified electronic screen combining an ICD-10 codelist and Chinese/English keywords. Encounters by the same child within 14 days were treated as one episode, and transfer-to-admission records were linked. Of 12,480 all-grade events, 452 were flagged for full review. The 12,028 screen-negative records were not subjected to systematic manual validation; therefore, screening sensitivity and negative predictive value could not be estimated, and missed institution-defined Grade III episodes cannot be excluded. Among reviewed charts, 75 had indeterminate pretreatment grade or timing and 35 did not meet institution-defined Grade III criteria, leaving 342 episodes meeting the local Grade III definition. Further exclusions were age outside criterion (*n* = 7), corticosteroid before the qualifying assessment (*n* = 25), and prespecified severe comorbidity (*n* = 10), leaving 300 eligible children; categories were mutually exclusive. The codelist, keyword list, event-linkage rules, and data-processing log were archived before analysis and are available from the corresponding author for editorial or independent review. Potential coding/documentation changes were considered in calendar-time analyses, but residual ascertainment effects cannot be excluded.

### 2.4 Eligibility, severity pathway, and differential diagnoses

Eligibility required age younger than 12 years, hospitalization, a qualifying institution-defined Grade III assessment before any epinephrine or systemic or inhaled corticosteroid, and initiation of one study corticosteroid strategy with epinephrine within a prespecified ±15-minute analytic window. Children could have Grade III documented at the first assessment at this center or progress from Grade II to Grade III after arrival before treatment. For transferred children, the first assessment at this center was not assumed to be the first medical assessment of the illness; outside-hospital severity trajectories were classified as unknown when source records were unavailable. Grade III was a hospital-specific severity label. The institutional Grade III label was informed by the four-grade laryngeal-obstruction framework traditionally used in Chinese pediatric teaching, in which grades I-IV describe progressively worsening inspiratory obstruction (14). Its local implementation was institution-specific and was not assumed to be equivalent to an internationally validated croup severity category. The local implementation has not undergone formal external validation against the WCS or another international croup score. The institutional grade and reconstructed WCS were therefore analyzed separately with no numerical mapping. For retrospective abstraction, Grade III required stridor at rest plus marked retractions or clearly increased work of breathing, with or without agitation, reduced air entry, hypoxemia, or altered consciousness. Treatment and admission decisions were not part of the definition; the same criteria were applied before treatment assignment was reviewed. Prespecified alternative causes of upper-airway obstruction and severe comorbidities were excluded. All included children were younger than 60 months.

### 2.5 Time zero and treatment strategies

Eligibility time and all baseline severity measurements were fixed at the qualifying Grade III assessment before epinephrine or corticosteroid administration. The treatment question was the initial corticosteroid choice among children receiving epinephrine as common background treatment. *A* = 1 denoted budesonide and *A* = 0 denoted nebulized dexamethasone. Corticosteroid administration was time zero; epinephrine had to begin from 15 min before to 15 min after time zero under the prespecified treatment-window definition. Emergency-department arrival, qualifying assessment, admission decision, epinephrine administration, and corticosteroid administration were retained as separate timestamps. Thirty-seven children received epinephrine before corticosteroid time zero. The exact epinephrine-to-corticosteroid interval was included in timing analyses, and exclusion of pre-index epinephrine was a key sensitivity analysis. The primary analysis followed the initial-treatment-policy strategy; six later crossovers remained in their initial groups.

Budesonide inhalation suspension (1 mg/2 mL) was administered at a nominal dose of 2 mg (4 mL) per treatment via a jet nebulizer with a face mask. No additional diluent was used. Driving-gas flow was 5–6 L/min and treatment lasted approximately 10–15 min. When clinically indicated for persistent or recurrent stridor at rest, one repeat dose was permitted after a minimum interval of 20 min, with no more than two doses within 24 h.

Dexamethasone sodium phosphate injection (5 mg/mL) was nebulized under a local institutional protocol at 0.30 mg/kg (maximum 10 mg), diluted with 0.9% saline to 4 mL, and delivered by jet nebulizer with a face mask at 5–6 L/min for 10–15 min; at most one repeat dose was permitted within 24 h. The protocol provided a local inhaled-corticosteroid option for marked upper-airway obstruction. Nebulization was not an approved route in the injectable product labeling and therefore was off-label. It was administered separately from epinephrine and is not equivalent to standard oral, intramuscular, or intravenous dexamethasone.

L-epinephrine 1 mg/mL (1:1,000) was administered by nebulization at 0.5 mL/kg per dose, to a maximum of 5 mL, separately from the corticosteroid. Repeat dosing was permitted for persistent or recurrent stridor at rest with increased work of breathing, at a minimum interval of 20 min under continuous cardiorespiratory monitoring. The number of doses, dose intervals, sequence relative to corticosteroid treatment, and time from the final dose to disposition were recorded.

### 2.6 Data extraction and reliability

Pretreatment covariates were abstracted at the qualifying Grade III assessment before epinephrine or corticosteroid administration. Variables included age, sex, calendar year, season, severity pathway at this center, reconstructed WCS, peripheral oxygen saturation (SpO_2_), fever duration, oxygen use, previous croup, C-reactive protein (CRP), white blood cell (WBC) count, neutrophil percentage, interhospital transfer, and pre-index epinephrine. Emergency-department-arrival-to-Grade-III, Grade-III-to-corticosteroid, and epinephrine-to-corticosteroid intervals were retained as timing variables. Patient identifiers were used to detect repeat episodes. All 300 included episodes were from unique children, and no pretreatment covariate was missing.

Thirty charts (10%) were independently double-abstracted across treatment groups and calendar periods. The reconstructed WCS followed the original scoring framework (15). The intraclass correlation coefficient [ICC (2,1), absolute agreement] for total reconstructed WCS was 0.89 (95% CI 0.78–0.95). Abstractors received standardized training, discrepancies were adjudicated by a senior pediatrician, and chart review followed recommended retrospective abstraction principles (16).

### 2.7 Outcomes

The primary outcome was time from corticosteroid time zero to the first physician or nursing assessment documenting no stridor at rest, with no recurrence at the next assessment within 60 min. During the first 12 h, airway status was generally documented approximately hourly. Assessment timing was not protocolized, and retrospective records did not permit a reliable comparison of clinical assessment frequency between treatment groups or across calendar years during the study period. Because resolution was interval-censored, the records bracketing disappearance were retained; estimates represent documented rather than continuously observed resolution.

Secondary clinical outcomes were baseline-adjusted 24-hour WCS, length of stay, repeat epinephrine, and post-index oxygen therapy. These outcomes used available-case data; missing outcome values were not imputed. ICU transfer, intubation, and death were rare safety events reported descriptively, without comparative inference. Six- and 12-month recurrence and billing were exploratory; recurrence used hospital-system records only, with denominators reported when incomplete. Hospital billing was descriptive, standardized to 2023 CNY, and not a cost-effectiveness analysis, with reporting informed by general health-economic methods (17, 18).

### 2.8 Estimand and statistical analysis

The target population was eligible children at this center who received epinephrine within the defined grace window and initiated one of the two study corticosteroid strategies. The contrast was initial budesonide (*A* = 1) versus initial nebulized dexamethasone (*A* = 0) on a treatment-policy basis, and the primary estimand was the sample average treatment effect of corticosteroid choice on hours to documented stridor resolution. Treatment switching after time zero did not redefine exposure. This estimand concerns corticosteroid choice under a common epinephrine background, not two fully separable combination regimens.

A logistic propensity-score model included variables available before corticosteroid assignment: age, sex, qualifying-assessment WCS, calendar year, season, qualifying-assessment SpO_2_, fever duration, baseline oxygen use, prior croup, CRP, WBC count, neutrophil percentage, severity pathway at this center, transfer, and pre-index epinephrine. Stabilized average-treatment-effect (ATE) weights were w = A·P(A = 1)/e(X) + (1−A)·P(A = 0)/[1−e(X)]. Values below the 1st and above the 99th percentiles were replaced with pooled empirical cutoffs using linear-interpolation quantiles (winsorization, not deletion). Robust standard errors were used. Diagnostics included treatment-specific propensity-score and pre/post-winsorization weight quantiles, common support, tail contribution, group-specific and overall effective sample size (ESS), standardized mean differences (SMDs), variance ratios, and a complete balance table (19–23).

Sensitivity analyses used unmodified weights, 2.5th/97.5th-percentile winsorization, conventional covariate regression, augmented inverse probability weighting (AIPW), categorical calendar year, restriction to calendar years 2017–2023, Grade III documented at the first assessment at this center, non-transfers, no epinephrine before corticosteroid time zero, continuous adjustment for the epinephrine-to-corticosteroid interval, empirical common-support restriction, and first eligible episode per patient. Each restricted analysis refitted the propensity model and weights. These analyses were *post hoc* unless otherwise specified. Overlap weighting, if used, was treated as an average treatment effect in the overlap population rather than as validation of the ATE.

For AIPW, the unmodified propensity score was combined with separate linear outcome regressions for each treatment arm containing the same covariates as the propensity model. The ATE was the mean augmented influence-function contribution, and its 95% CI used the empirical influence-function standard error. Cross-fitting was not used. Double robustness was interpreted as conditional on correct specification of at least one nuisance model and not as evidence that either model was verified (21, 24).

Binary outcomes were summarized with raw n/N and weighted risk differences; rare airway outcomes were descriptive. Secondary and exploratory outcomes were not interpreted confirmatorily; recurrence was not assigned causal interpretation. No formal *a priori* sample-size calculation was performed; all children meeting eligibility after the prespecified screening/chart-review process were included. Group sizes were not treatment-stratified. Billing uncertainty used robust methods for skewed outcomes. Analyses used Python 3.12.13, NumPy 2.3.5, and pandas 3.0.1; full details are in Supplementary Methods.

### 2.9 Targeted literature search

To update the clinical background, we conducted a targeted, non-systematic search on 10 August 2026 in PubMed/MEDLINE, the Cochrane Library, and the online croup guidance repositories of the Canadian Paediatric Society and the Royal Children's Hospital Melbourne. Eligible records were pediatric croup studies comparing corticosteroid agents, doses, routes, or schedules; systematic reviews; and current clinical guidance. Full search strings, date limits, eligibility criteria, and screening procedures are reported in Supplementary Methods S1. The search was not registered or conducted as a systematic review.

## 3 Results

### 3.1 Cohort assembly and timing

Of 12,480 all-grade croup presentation events, 452 were flagged for full chart review. After exclusion of 75 records with indeterminate pretreatment grade/timing and 35 not meeting institution-defined Grade III criteria, 342 met the local Grade III definition (minimum confirmed proportion, 2.74%). Screen-negative records were not manually validated; thus this value is not a prevalence estimate, and missed Grade III episodes remain possible.

Further exclusions were age outside criterion (*n* = 7), corticosteroid before the qualifying assessment (*n* = 25), and prespecified severe comorbidity (*n* = 10), leaving 300 unique children. All eligible children identified by the prespecified screen and chart review were included without treatment-stratified sampling; 150 received each strategy. The institution-defined Grade III label was documented at first assessment in 264 children (88.0%); 36 (12.0%) progressed from Grade II after arrival, and 84 (28.0%) were interhospital transfers (Figure 1). Outside-hospital severity at first contact and pre-transfer Grade II-to-III evolution were unknown when records were unavailable.

**Figure 1:**
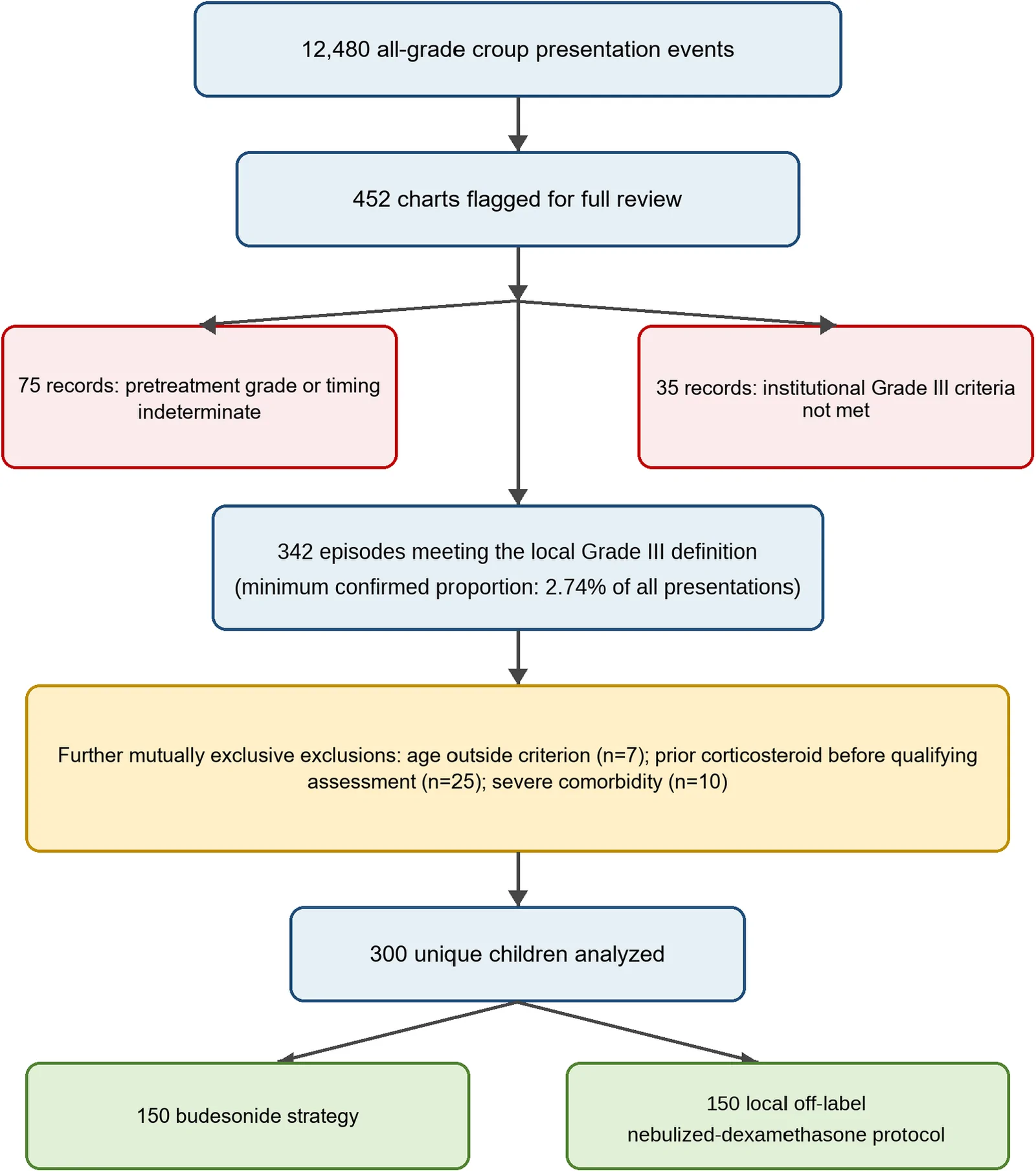
Screen-negative records (*n* = 12,028) were not subjected to systematic manual validation; missed institution-defined Grade III episodes, and selection bias cannot be excluded.

Median time from ED arrival to qualifying Grade III assessment was 0.18 h (IQR 0.15–0.23) among children Grade III at the first hospital assessment and 1.63 h (IQR 1.20–1.82) among those progressing from Grade II. Median qualifying-assessment-to-steroid time was 10.9 min (IQR 8.5–14.5).

### 3.2 Baseline characteristics

Baseline characteristics, propensity-score and weighting diagnostics, primary and sensitivity analyses, short-term outcomes, recurrence, and hospital billing results are summarized in Tables 1–6, respectively. Calendar year had the largest raw imbalance (absolute SMD 0.588); annual counts (Table 7) showed marked temporal separation, with greatest overlap in 2022–2023. Dexamethasone recipients also had higher WCS, lower oxygen saturation, more oxygen use, longer fever duration, and more transfers, reinforcing concern about secular and indication-related confounding.

**Table 1:** Raw pretreatment characteristics.

Variable	Budesonide + epinephrine (*n* = 150)	Nebulized dexamethasone + epinephrine (*n* = 150)	Raw SMD
Age, months	24.60 ± 5.00	24.90 ± 5.20	−0.059
Male sex, *n* (%)	75 (50.0)	74 (49.3)	0.013
Reconstructed WCS	6.50 ± 1.10	6.80 ± 1.20	−0.261
Calendar year	2020.10 ± 2.70	2018.30 ± 3.40	0.588
Winter/spring, *n* (%)	93 (62.0)	78 (52.0)	0.203
SpO_2_, %	94.20 ± 1.80	93.50 ± 2.00	0.368
Fever duration, h	18.00 ± 10.50	22.40 ± 13.20	−0.369
Oxygen use at qualifying Grade III assessment, *n* (%)	43 (28.7)	61 (40.7)	−0.254
Previous croup, *n* (%)	18 (12.0)	24 (16.0)	−0.116
CRP, mg/L	10.00 ± 3.60	11.20 ± 4.10	−0.311
WBC, ×10^9^/L	8.40 ± 2.00	8.80 ± 2.10	−0.195
Neutrophils, %	63.80 ± 6.90	65.60 ± 7.40	−0.252
Progressed Grade II-to-III, *n* (%)	20 (13.3)	16 (10.7)	0.082
Interhospital transfer, *n* (%)	36 (24.0)	48 (32.0)	−0.179
Epinephrine before steroid, *n* (%)	17 (11.3)	20 (13.3)	−0.061

Values are unweighted mean ± SD or *n* (%). WCS, Westley Croup Score; SMD, standardized mean difference; SpO_2_, peripheral oxygen saturation; CRP, C-reactive protein; WBC, white blood cell count. Grade III refers to the local institutional classification.

**Table 2:** Treatment-specific propensity-score and stabilized-weight distributions before and after winsorization.

Quantile	PS: bud	PS: dex	Raw w: bud	Raw w: dex	Post w: bud	Post w: dex
Minimum	0.078	0.019	0.512	0.510	0.523	0.523
1st percentile	0.162	0.034	0.534	0.518	0.534	0.523
5th percentile	0.274	0.068	0.555	0.536	0.555	0.536
Median	0.643	0.356	0.777	0.776	0.777	0.776
95th percentile	0.902	0.780	1.824	2.275	1.824	2.275
99th percentile	0.936	0.855	3.265	3.454	3.091	3.454
Maximum	0.976	0.893	6.423	4.683	3.671	3.671
Mean	—	—	0.972	1.009	0.951	1.003

PS, propensity score; w, stabilized inverse-probability-of-treatment weight; bud, budesonide; dex, nebulized dexamethasone. Post-winsorization values use pooled empirical 1st/99th-percentile cutoffs.

**Table 3:** Primary-outcome and sensitivity analyses.

Analysis	*n*	Difference, h	95% CI	Estimand/note
Crude	300	−2.65	−2.96 to −2.34	Unweighted
Conventional regression	300	−2.23	−2.54 to −1.92	Post hoc
IPTW, unmodified	300	−2.24	−2.61 to −1.87	Unmodified sample-ATE estimator
IPTW, 1st/99th winsorized	300	−2.26	−2.63 to −1.89	Primary winsorized ATE-weight estimator; approximation to sample ATE
IPTW, 2.5th/97.5th winsorized	300	−2.30	−2.65 to −1.94	Post hoc winsorized ATE-weight estimator
AIPW, unmodified PS	300	−2.24	−2.55 to −1.93	Post hoc ATE
Calendar year categorical	300	−2.32	−2.71 to −1.94	Post hoc ATE
Calendar-period restriction, 2017–2023	177	−2.23	−2.65 to −1.80	Post hoc restriction
Grade III at first center assessment	264	−2.28	−2.68 to −1.88	Post hoc restriction
Non-transfer	216	−2.33	−2.76 to −1.90	Post hoc restriction
No epinephrine before steroid	263	−2.27	−2.63 to −1.90	Key *post hoc* restriction
Continuous epinephrine→steroid interval adjustment	300	−2.27	−2.63 to −1.91	Post hoc weighted outcome model
Empirical common-support restriction	280	−2.23	−2.60 to −1.87	Post hoc restricted-population effect
First eligible episode	300	−2.26	−2.63 to −1.89	All IDs unique

ATE, average treatment effect; IPTW, inverse probability of treatment weighting; AIPW, augmented inverse probability weighting; PS, propensity score. Negative differences favor budesonide for time to documented stridor resolution. Grade III denotes the institution-defined local severity classification.

**Table 4:** Short-term outcomes.

Outcome	Raw budesonide summary	Raw dexamethasone summary	Adjusted/weighted effect
24-h WCS	2.30 ± 0.95	3.21 ± 1.00	Adjusted difference −0.85 (−1.09 to −0.61)
Length of stay, days	4.80 ± 1.20	6.10 ± 1.50	Difference −1.24 (−1.57 to −0.91)
Repeat epinephrine, n/N	33/150	45/150	Weighted RD −5.2% (−16.7 to 6.3)
Post-index oxygen, n/N	46/150	52/150	Weighted RD −5.5% (−17.5 to 6.5)
ICU transfer, n/N	4/150	6/150	Descriptive only
Intubation, n/N	0/150	1/150	Descriptive only
Death, n/N	0/150	0/150	Descriptive only

Group summaries are unweighted raw mean ± SD or n/N. WCS, Westley Croup Score; RD, risk difference; ICU transfer, intubation, and death are descriptive only and do not support safety equivalence.

**Table 5:** Exploratory recurrence outcomes.

Outcome	Budesonide	Nebulized dexamethasone	Interpretation
6-month recurrence	17/138 (12.3%)	29/130 (22.3%)	Descriptive; incomplete system visibility
12-month recurrence	23/125 (18.4%)	33/115 (28.7%)	Descriptive; incomplete system visibility

Recurrence was visible only within the hospital information system. Denominators represent children with observable follow-up and are not assumed to be non-informatively missing.

**Table 6:** Exploratory hospital billing standardized to 2023 CNY.

Billing category	Raw budesonide mean ± SD	Raw dexamethasone mean ± SD	IPTW-weighted mean difference (95% CI)
Drug billing	306.00 ± 50.00	397.00 ± 58.00	−90 (−104 to −77)
Other hospitalization billing	2520.00 ± 310.00	2775.00 ± 355.00	−266 (−349 to −182)
Total direct hospital billing	2826.00 ± 317.12	3172.00 ± 362.59	−356 (−441 to −272)

CNY, Chinese yuan; IPTW, inverse probability of treatment weighting; CI, confidence interval. Amounts were standardized to 2023 CNY and represent descriptive hospital billing outcomes, not a cost-effectiveness analysis.

**Table 7:** Distribution of initial corticosteroid strategy by calendar year.

Calendar year	Budesonide, n	Local off-label nebulized dexamethasone, n	Total, n
2014	1	1	2
2015	10	38	48
2016	24	49	73
2017	1	1	2
2018	1	1	2
2019	1	1	2
2020	26	1	27
2021	28	2	30
2022	29	27	56
2023	29	29	58
Total	150	150	300

Values are unweighted observed counts. The *post hoc* calendar-period restriction to 2017–2023 included 177 children: 115 in the budesonide group and 62 in the local off-label nebulized-dexamethasone group.

### 3.3 Propensity-score and weighting diagnostics

Propensity scores ranged from 0.019 to 0.976, with central overlap and sparse tails. The empirical common-support interval was 0.078 to 0.893; 10 budesonide and 10 dexamethasone observations fell outside it. Raw stabilized weights had mean 0.991, median 0.777, and maximum 6.423; the three largest weights contributed 5.1% of the total raw weight. Six weights (2.0%) were replaced at pooled cutoffs 0.523 and 3.671; the post-winsorization maximum was 3.671. Overall ESS was 226.3 (budesonide 117.5; dexamethasone 109.6). The maximum post-weighting absolute SMD was 0.076 (Figure 2) and post-weighting variance ratios ranged from 0.677 to 1.082. The propensity-score and post-winsorization stabilized-weight distributions are shown in Figure 3. Complete balance diagnostics are reported in Supplementary Table S1, with the Love plot and propensity-score/weight distributions in Supplementary Figures S1 and S2.

**Figure 2:**
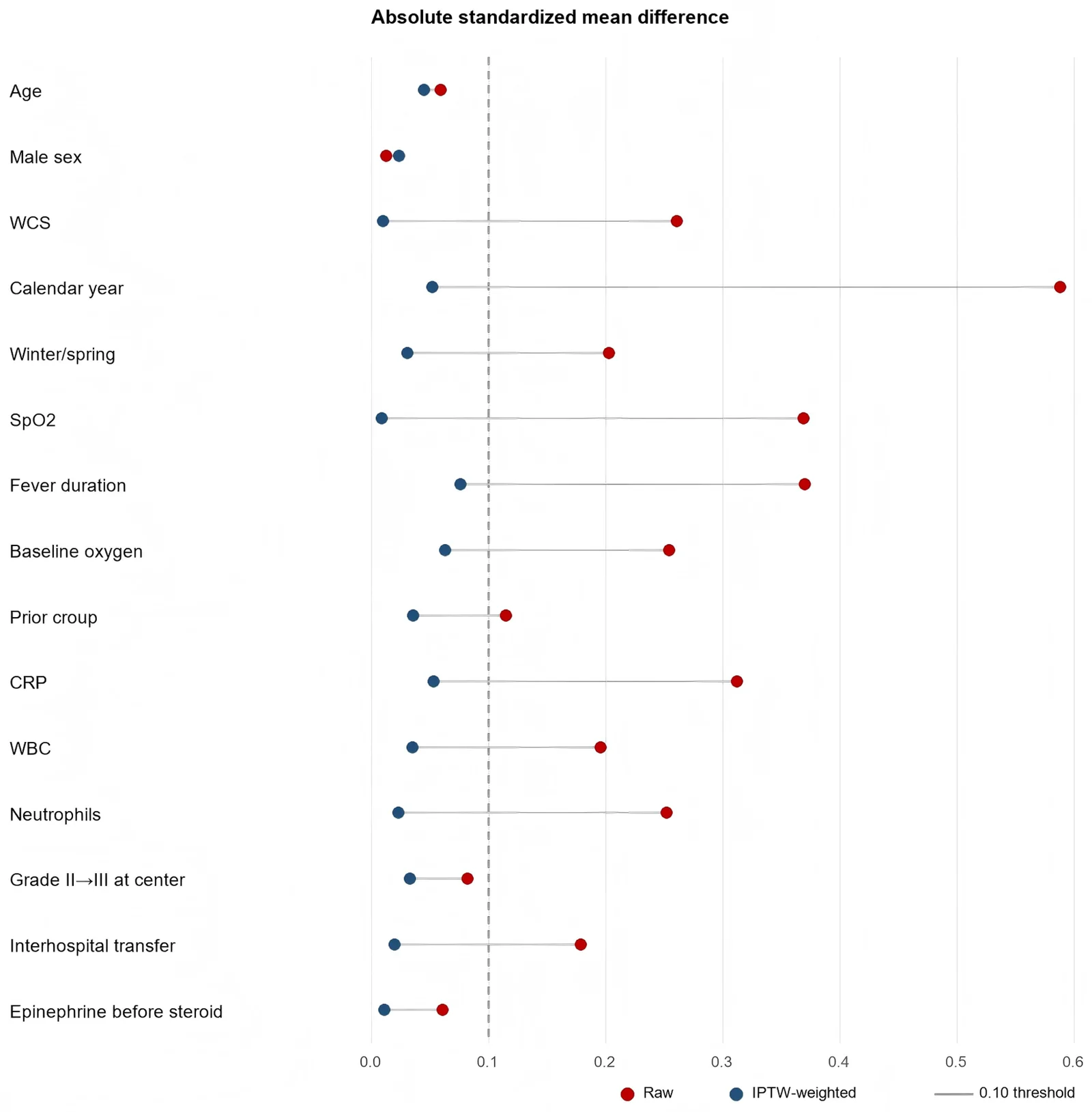
Absolute standardized mean difference.

**Figure 3:**
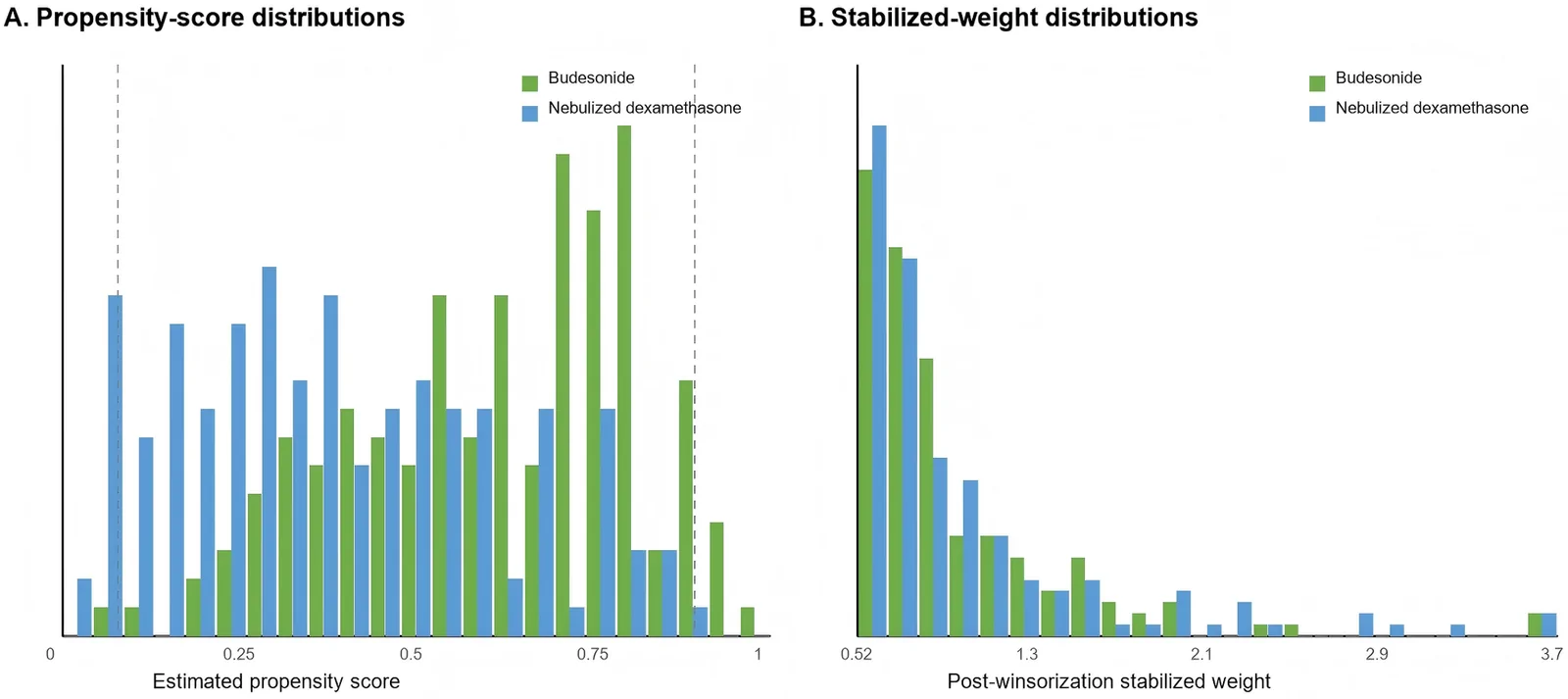
**(A)** Propensity-score distributions. **(B)** Stabilized-weight distributions.

### 3.4 Primary outcome and sensitivity analyses

Raw mean time to documented stridor resolution was 6.96 ± 1.25 h versus 9.60 ± 1.48 h. The crude difference was −2.65 h (95% CI −2.96 to −2.34). After 1st/99th-percentile ATE weighting, weighted means were 7.05 and 9.31 h, and the difference was −2.26 h (95% CI −2.63 to −1.89).

Effect estimates were directionally consistent across the specified sensitivity analyses. This consistency indicates limited sensitivity to the examined modeling and restriction choices but does not exclude residual confounding or model misspecification.

### 3.5 Secondary clinical outcomes

Ten ICU transfers occurred. In the budesonide group, three were primarily attributed to persistent or worsening upper-airway obstruction and one to hypoxemia or respiratory fatigue; in the nebulized-dexamethasone group, five were attributed to upper-airway obstruction and one to hypoxemia or respiratory fatigue. None was primarily attributed to a comorbidity or bed-availability reason. The sole intubation occurred in the dexamethasone group 2.5 h after corticosteroid time zero for progressive upper-airway obstruction with respiratory fatigue and hypoxemia. Mechanical ventilation lasted 36 h, followed by extubation and recovery without documented airway sequelae. No deaths occurred. These event counts are too sparse to support comparative safety conclusions or safety equivalence.

### 3.6 Exploratory recurrence and hospital billing outcomes

Recurrence denominators were lower than the analytic cohort and ascertainment was limited to the hospital information system; no causal inference was made. Billing differences were not interpreted as cost-effectiveness and may partly reflect length of stay, secular price changes, and institutional billing practice.

## 4 Discussion

In this single-center cohort, the initial budesonide strategy was associated with a 2.26-hour shorter documented time to stridor resolution after weighting. Measured balance improved, but ESS fell from 300 to 226.3 and sparse propensity-score tails remained. Twenty observations lay outside empirical common support; therefore, the full-sample ATE-weight analysis depends partly on propensity-model extrapolation in the tails, and the common-support restriction represents a distinct restricted-population analysis. Winsorization limits the influence of extreme weights but does not restore empirical positivity. These findings support an adjusted association and do not establish clinical superiority.

### 4.1 Relation to current evidence

The 2023 Cochrane review supports glucocorticoids as a class but does not establish superiority of the local regimen studied here (2). Earlier randomized comparisons generally used oral or intramuscular dexamethasone, while Luria et al. evaluated nebulized dexamethasone in mild croup and favored oral dexamethasone for some outcomes (6). Current Canadian and Australian guidance describes oral dexamethasone as standard therapy and does not present nebulized dexamethasone as a standard route (10, 11). The comparator here was a local institutional protocol using off-label nebulization of dexamethasone sodium phosphate injection; nebulization was not an approved route in the product labeling and should not be considered equivalent to standard oral, intramuscular, or intravenous dexamethasone. Recent studies addressed other corticosteroid questions rather than this route-specific comparison (7–9). Our targeted non-systematic search identified no contemporary study of this exact comparison.

### 4.2 Residual confounding and calendar time

Treatment by indication remains plausible: clinicians may have selected dexamethasone for children who appeared sicker in undocumented ways. Annual counts (Table 7) show strong temporal imbalance: dexamethasone predominated in 2015–2016, budesonide in 2020–2021, and overlap was greatest in 2022–2023. Calendar-year adjustment and the 2017–2023 calendar-period restriction were directionally consistent; however, neither can remove unmeasured secular changes in referral patterns, drug supply, documentation, admission thresholds, or COVID-era practice. Residual long-term confounding therefore remains important and cannot be quantified from the available records.

### 4.3 Time zero and outcome measurement

Defining corticosteroid administration as time zero clarifies the treatment contrast but does not eliminate bias from epinephrine given immediately beforehand. Epinephrine can rapidly improve stridor and may influence subsequent corticosteroid selection. We therefore retained the exact epinephrine-to-corticosteroid interval and performed analyses excluding pre-index epinephrine and adjusting for the continuous interval. As assessment timing was not protocolized, retrospective records could not establish comparable clinical assessment frequency between groups or across calendar years during the study period. Differential assessment or documentation frequency may have biased recorded resolution times in an unknown direction; estimates represent documented rather than continuous resolution.

### 4.4 Generalizability and treatment route

A provincial tertiary pediatric center may receive a disproportionate number of high-acuity and transferred cases. In this cohort, 28.0% were interhospital transfers, but only 10/300 required ICU transfer, 1/300 required intubation, and no deaths occurred. The cohort therefore represents high-risk hospitalized children with significant upper-airway obstruction rather than uniformly PICU-level critical illness. Findings apply to this local Grade III definition and this institutional off-label nebulized-dexamethasone protocol, not to a universal severe-croup category or standard systemic dexamethasone therapy.

### 4.5 Rare events, recurrence, and billing

ICU transfer (4/150 vs. 6/150), intubation (0/150 vs. 1/150), and death (0/150 vs. 0/150) were too sparse for comparative safety inference; absence of deaths or non-significant differences does not demonstrate safety equivalence. Recurrence was incompletely observed outside the hospital system and remains descriptive. Hospital billing is exploratory, not a cost-effectiveness analysis, and may reflect length of stay.

### 4.6 Strengths and limitations

Strengths include explicit accounting of the all-grade source population, a clearly defined pretreatment severity assessment, medication-administration timestamps, separate reporting of within-center Grade II-to-III progression and interhospital transfer, and comprehensive propensity-score diagnostics.

Limitations include the retrospective single-center design; a nonstandard local Grade III classification without external validation; screen-negative records were not manually validated, so missed cases and potential selection bias cannot be excluded; an unusual off-label local nebulized-dexamethasone protocol; possible treatment-by-indication and secular confounding; exclusion of records with unresolved pretreatment grade/timing; interval-censored outcome measurement, non-protocolized assessment timing, and inability to retrospectively confirm comparable clinical assessment frequency between treatment groups or across calendar years during the study period; cost skewness and price-year effects; and limited information for rare harms. All children meeting eligibility after the prespecified screening and chart-review process were included; group sizes were not treatment-stratified. Secondary/exploratory analyses were not multiplicity-adjusted and require caution.

## 5 Conclusion

Among hospitalized children receiving epinephrine for institution-defined Grade III croup, initial nebulized budesonide was associated with faster documented stridor resolution than the local institutional off-label nebulized-dexamethasone protocol after adjustment for measured pretreatment variables. This comparison does not address standard oral, intramuscular, or intravenous dexamethasone therapy. Residual confounding, sparse propensity-score tails, interval-censored outcome measurement, and the unusual off-label comparator route limit causal and external interpretation; the findings remain adjusted observational associations rather than evidence of treatment superiority.

## Data Availability

The datasets generated and analyzed during the study are not publicly available because they contain potentially identifiable pediatric clinical information and are subject to institutional and ethical restrictions. A de-identified data dictionary and analysis code are available from the corresponding author upon reasonable request and with approval from the Ethics Committee of Jiangxi Provincial Children's Hospital. Requests to access the datasets should be directed to Yixian Yang, 76526012@qq.com.

## Footnotes

^1^Canadian Paediatric Society guideline: https://cps.ca/en/documents/position/acute-management-of-croup (updated 6 March 2026; accessed 11 August 2026). Royal Children's Hospital Melbourne guideline: https://www.rch.org.au/clinicalguide/guideline_index/Croup_Laryngotracheobronchitis/ (last updated September 2024; accessed 11 August 2026).
