# Supplementary material for: Initial nebulized budesonide versus a local off-label nebulized dexamethasone protocol in children with institution-defined grade III croup receiving epinephrine: a retrospective cohort study
Source: Front Pediatr. 2026 Sep 23;14:1948559. doi: 10.3389/fped.2026.1948559 (PMC13642681; doi:10.3389/fped.2026.1948559)
Supplement: Supplementary file 1 [file Datasheet1.pdf]

Supplementary Material

Contents

- Supplementary Methods S1. Targeted literature search
- Supplementary Table S1. Complete covariate-balance diagnostics
- Supplementary Figure S1. Love plot
- Supplementary Figure S2. Propensity-score and stabilized-weight distributions
- SupplementaryTableS2.Analysis-variabledictionary
- Appendix A. Supplementary Methods: analysis reproducibility

Supplementary Methods S1. Targeted literature search

Search date: 10 August 2026. The search was targeted and non-systematic; it was not registered and was not intended to satisfy PRISMA requirements for a systematic review.

PubMed/MEDLINE search: (croup[Title/Abstract] OR laryngotracheobronchitis[Title/Abstract]) AND (budesonide[Title/Abstract] OR dexamethasone[Title/Abstract] OR prednisolone[Title/Abstract] OR prednisone[Title/Abstract] OR corticosteroid*[Title/Abstract] OR glucocorticoid*[Title/Abstract]) AND (random*[Title/Abstract] OR trial[Title/Abstract] OR cohort[Title/Abstract] OR comparative[Title/Abstract] OR systematic review[Publication Type] OR meta-analysis[Publication Type]). Date window for contemporary comparative studies: 1 January 2016 to 10 August 2026; no date restriction was applied when locating route-specific foundational trials.

Cochrane Library search: (croup OR laryngotracheobronchitis) AND (budesonide OR dexamethasone OR prednisolone OR glucocorticoid*). The most recent eligible review update was retained.

Guideline search: the Canadian Paediatric Society and Royal Children's Hospital Melbourne websites were searched for current croup guidance using the terms croup, dexamethasone, budesonide, nebulized steroid, and epinephrine.

Eligibility: pediatric croup populations; comparative corticosteroid agent, dose, route, or schedule; systematic reviews of glucocorticoids for croup; or current clinical guidance. Non-croup laryngitis, recurrent-croup etiology studies, and studies without a corticosteroid strategy comparison were excluded from the clinical-comparison narrative.

Screening and conclusion: records were screened for title/abstract relevance and full text was checked when eligibility or treatment route was uncertain. Because this was a targeted narrative search, no deduplicated PRISMA record count or risk-of-bias assessment was generated. The search identified recent evidence comparing standard- and low-dose dexamethasone with prednisolone, dexamethasone with prednisone, and single with multiple dexamethasone dosing, together with the 2023 Cochrane update. It did not identify an eligible contemporary study directly comparing nebulized budesonide with nebulized dexamethasone in hospitalized Grade III croup. This negative finding is limited to the stated targeted search and should not be interpreted as proof that no such study exists.

Supplementary Table S1. Complete covariate-balance diagnostics

Covariate	Raw SMD	Weighted SMD	Raw variance ratio	Weighted variance ratio
Age	−0.059	−0.045	0.925	0.966
Male sex	0.013	0.024	1.000	0.997
WCS	−0.261	0.010	0.840	1.023
Calendar year	0.588	0.052	0.631	0.783
Winter/spring	0.203	0.031	0.944	0.992
SpO_2	0.368	0.009	0.810	0.833
Fever duration	−0.369	−0.076	0.633	0.677
Baseline oxygen	−0.254	−0.063	0.847	0.953

Covariate	Raw SMD	Weighted SMD	Raw variance ratio	Weighted variance ratio
Prior croup	−0.116	−0.036	0.786	0.922
CRP	−0.311	−0.053	0.771	0.725
WBC	−0.195	−0.035	0.907	0.876
Neutrophils	−0.252	−0.023	0.869	0.867
Grade II-to-III at center	0.082	0.033	1.213	1.082
Interhospital transfer	−0.179	−0.020	0.838	0.979
Epinephrine before steroid	−0.061	0.011	0.870	1.025

Note. SMD, standardized mean difference. Variance ratios compare budesonide with nebulized dexamethasone.

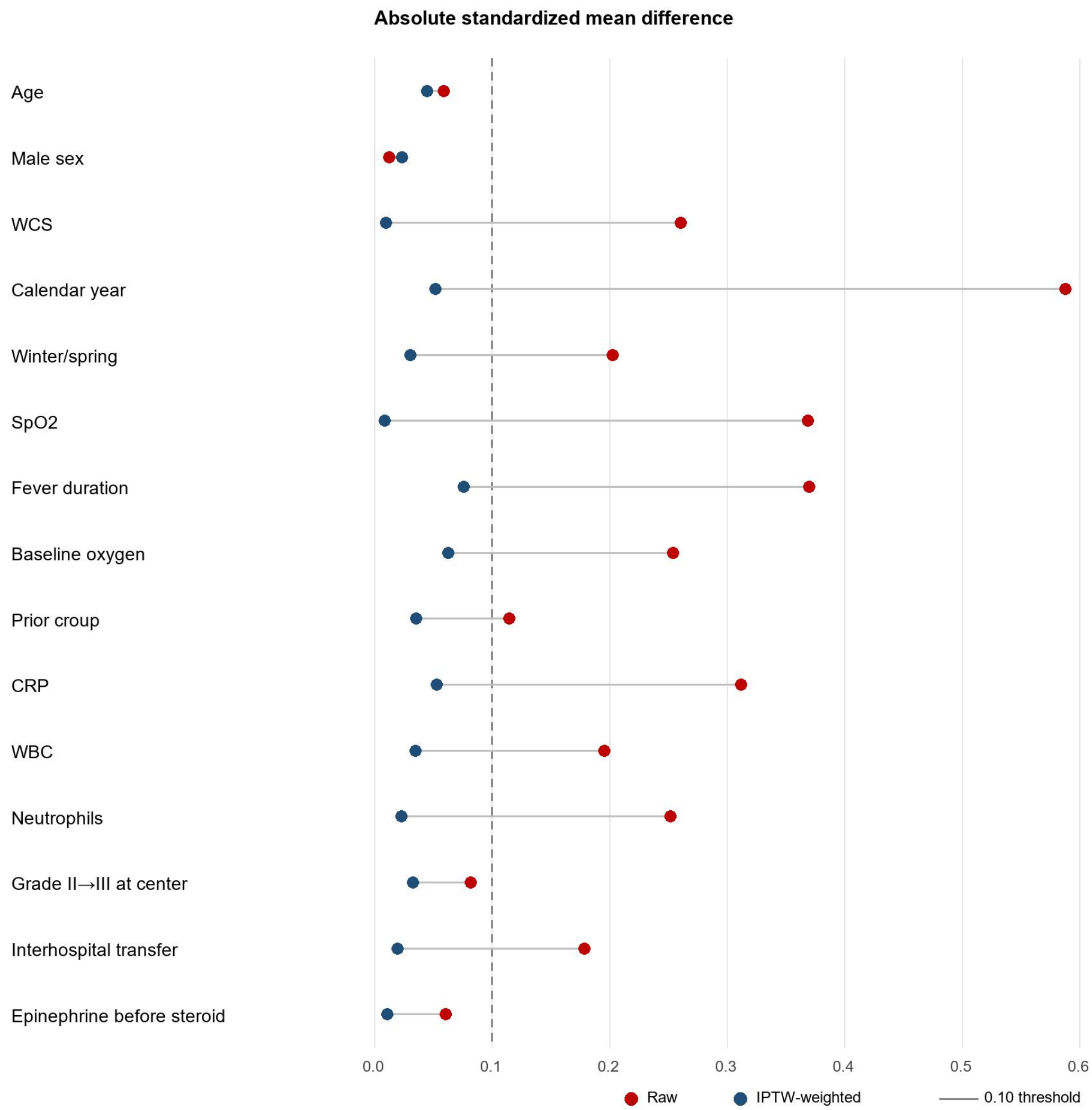

Supplementary Figure S1. Love plot for raw and IPTW-weighted covariate balance

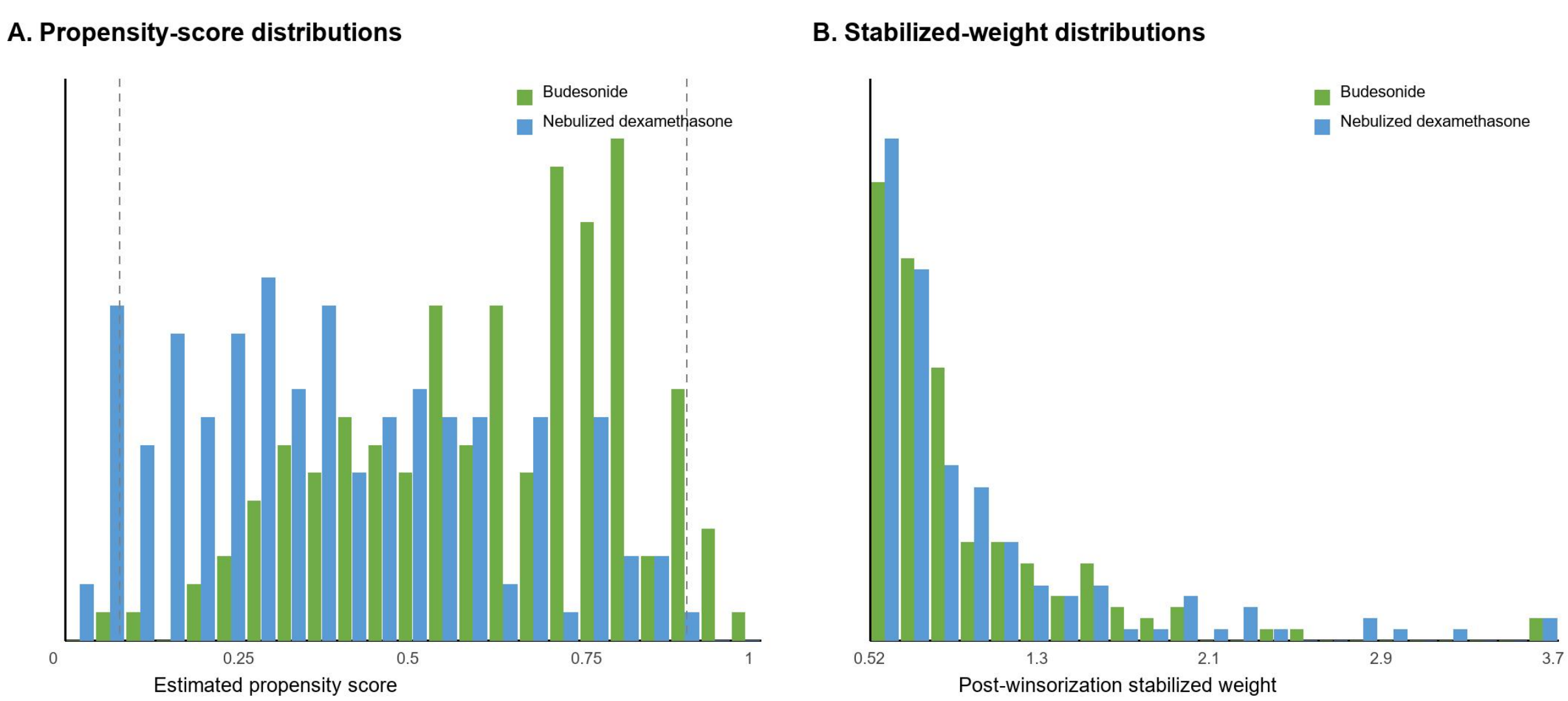

Supplementary Figure S2. Propensity-score and stabilized-weight distributions by treatment strategy

Supplementary Table S2. Analysis-variable dictionary

Variable	Definition	Type	Coding/units
A	Initial corticosteroid strategy (A = 1, nebulized budesonide; A = 0, nebulized dexamethasone)	Binary/integer	0/1
age	Age at qualifying assessment, months	Continuous	months; ≥0
male	Male sex indicator	Binary/integer	0/1
wcs	Reconstructed pretreatment Westley Croup Score	Integer	integer total score
year	Calendar year of episode	Integer	integer 2014–2023
season	Winter/spring season indicator	Binary/integer	0/1
spo2	Pretreatment peripheral oxygen saturation, %	Continuous	%
fever	Fever duration before qualifying assessment, h	Continuous	hours; ≥0
oxygen	Oxygen use at qualifying Grade III assessment	Binary/integer	0/1
prior	Previous croup indicator	Binary/integer	0/1
crp	C-reactive protein, mg/L	Continuous	mg/L; ≥0
wbc	White blood cell count, $\times 10^9$/L	Continuous	$\times 10^9$/L; ≥0
neut	Neutrophil percentage	Continuous	%
progress	Progressed from Grade II to Grade III after arrival at this center	Binary/integer	0/1
transfer	Interhospital transfer indicator	Binary/integer	0/1
preepi	Epinephrine began before steroid time zero	Binary/integer	0/1
ps	Estimated propensity score for budesonide	Continuous	0–1
stridor_h	Hours from steroid time zero to documented resolution of stridor at rest	Continuous	hours; ≥0
sw_raw	Raw stabilized ATE weight	Continuous	positive continuous
sw	Post-winsorization stabilized ATE weight	Continuous	positive continuous
arrival_to_grade_h	Hours from emergency-department arrival to qualifying Grade III assessment	Continuous	hours; ≥0
grade_to_steroid_min	Minutes from qualifying Grade III assessment to steroid time zero	Continuous	minutes; ≥0
epi_minus_steroid_min	Epinephrine start minus steroid start, minutes; negative values indicate pre-index epinephrine	Continuous	minutes; negative=pre-index
wcs_24h	Reconstructed WCS at 24 h	Integer	integer total score
wcs_improvement	Baseline WCS minus 24-h WCS	Integer	integer difference
los_days	Hospital length of stay, days	Continuous	days; ≥0
repeat_epi	Repeat epinephrine indicator	Binary/integer	0/1
oxygen_after	Post-index oxygen therapy indicator	Binary/integer	0/1
icu	ICU transfer indicator	Binary/integer	0/1
intubation	Endotracheal intubation indicator	Binary/integer	0/1
drug_cost_2023_cny	direct drug billing standardized to 2023 CNY	Continuous	2023 CNY; ≥0
hospital_cost_2023_cny	other hospital billing standardized to 2023 CNY	Continuous	2023 CNY; ≥0

Variable	Definition	Type	Coding/units
total_cost_2023_cny	total direct hospital billing standardized to 2023 CNY	Continuous	2023 CNY; ≥0
visible_6m	Observable in the hospital information system through 6 months	Binary/integer	0/1
recurrence_6m	Documented croup recurrence within 6 months among observable children	Binary/integer	0/1 among observable children
visible_12m	Observable in the hospital information system through 12 months	Binary/integer	0/1
recurrence_12m	Documented croup recurrence within 12 months among observable children	Binary/integer	0/1 among observable children
patient_id	unique patient identifier	Identifier/text	de-identified unique string
crossover_24h	Crossover to the alternative corticosteroid within 24 h	Binary/integer	0/1

Note. Variables are defined according to the analysis dataset. Pretreatment WCS and calendar year were stored as integer-valued variables; duration variables were constrained to non-negative values.

Appendix A. Supplementary Methods: analysis reproducibility

The analytic dataset contained 300 unique children, with 150 in each initial corticosteroid strategy. All eligible children identified by the prespecified screen and chart review were included without treatment-stratified sampling. Progression from Grade II to Grade III occurred in 20 and 16 children, interhospital transfer in 36 and 48, and pre-index epinephrine in 17 and 20 children in the budesonide and dexamethasone groups, respectively.

The primary analysis estimated the sample average treatment effect using stabilized inverse probability of treatment weights. The propensity-score model, covariate definitions, winsorization thresholds, outcome models, and restricted analyses were implemented as specified in Methods 2.8. No outcome values were generated or imputed for the primary analysis.

The propensity score used penalized logistic iteratively reweighted least squares with ridge parameter 0.15 and the covariates listed in Methods 2.8. Primary confidence intervals used a heteroskedasticity-robust sandwich estimator. AIPW used unmodified propensity scores, arm-specific linear outcome regressions containing all propensity covariates, and an empirical influence-function standard error. Restricted analyses refitted the propensity model and recomputed 1st/99th-percentile winsorized weights.

Runtime environment: Python 3.12.13; NumPy 2.3.5; pandas 3.0.1; lxml 6.0.2; python-docx 1.2.0. Package versions and analysis definitions were frozen before final result generation.

Reproducibility files

The corresponding author maintains the analysis scripts, environment specification, data dictionary, and execution log. These materials are available for editorial or independent statistical review subject to institutional data-governance requirements.
